# Wartenberg’s Syndrome: A Case of Ultrasound Revealing a Diagnosis Missed by Electromyography With Hydrodissection Offering Pain Relief and Functional Recovery

**DOI:** 10.7759/cureus.88503

**Published:** 2025-07-22

**Authors:** Mohamed Maroc, Abderrahim Lachhab, Abdelilah Rhoul, Mohamed Harmouche, Ahmed Amine El Oumri

**Affiliations:** 1 Faculty of Medicine, Mohammed I University, Oujda, MAR; 2 Physical Medicine and Rehabilitation, Mohammed VI University Hospital, Oujda, MAR

**Keywords:** electromyography, hydrodissection, superficial radial nerve, ultrasound, wartenberg’s syndrome

## Abstract

Wartenberg’s syndrome, an entrapment neuropathy of the superficial radial nerve, frequently presents significant diagnostic challenges, often with normal electroneuromyography findings despite debilitating clinical symptoms. This case report details a patient experiencing severe post-traumatic neuropathic pain highly consistent with Wartenberg’s syndrome. High-resolution ultrasound proved pivotal in revealing focal nerve enlargement and confirming the diagnosis, which was missed by conventional electroneuromyography. Subsequent ultrasound-guided hydrodissection led to substantial and sustained pain relief and functional recovery. This case underscores the indispensable role of high-resolution ultrasound in diagnosing Wartenberg’s syndrome when traditional electrophysiological studies are inconclusive and demonstrates the effectiveness of hydrodissection as a minimally invasive therapeutic solution for managing this challenging neuropathic pain.

## Introduction

The superficial radial nerve (SRN), owing to its superficial anatomical course along the forearm, is particularly susceptible to compression and injury, often at specific entrapment sites [1]. Such pathology can lead to conditions such as Wartenberg’s syndrome, also known as cheiralgia paresthetica, a distinct entrapment neuropathy characterized by pain and sensory disturbances primarily involving the dorsum of the hand and digits, which may, in some cases of more proximal entrapment, extend to the distal dorsolateral forearm [2].

A critical diagnostic dilemma often arises because electroneuromyography (ENMG) studies, typically a cornerstone of nerve assessment, may yield normal results despite clear and debilitating clinical symptoms [3]. This is particularly true in Wartenberg’s syndrome, where the affected SRN is purely cutaneous. While sensory nerve conduction studies (NCS) are the appropriate test to evaluate this cutaneous nerve, they frequently fail to detect subtle or intermittent compressions, leading to normal findings despite significant clinical presentation. Importantly, needle electromyography (EMG), which assesses muscle activity, is typically normal in this purely sensory condition, serving primarily to rule out other differential diagnoses involving motor nerve or muscle pathology (e.g., more proximal radial nerve compressions affecting motor branches or primary muscle diseases), thus further complicating the electrophysiological diagnosis and highlighting the limitations of conventional ENMG in these specific cases.

In such instances, high-resolution ultrasonography (US) emerges as a crucial diagnostic tool, offering unparalleled anatomical insights into nerve pathology and subtle entrapment not detectable by conventional electrophysiological methods [4,5]. Furthermore, the advent of ultrasound-guided hydrodissection, particularly using 5% dextrose in water (D5W), presents a promising, minimally invasive therapeutic option for peripheral nerve entrapment syndromes [6]. This case report details a challenging presentation of Wartenberg’s syndrome following forearm trauma, characterized by severe neuropathic pain, normal ENMG findings, and a favorable response to US-guided D5W hydrodissection, thereby emphasizing the indispensable role of US in both precise diagnosis and targeted treatment.

## Case presentation

**Figure 1:**
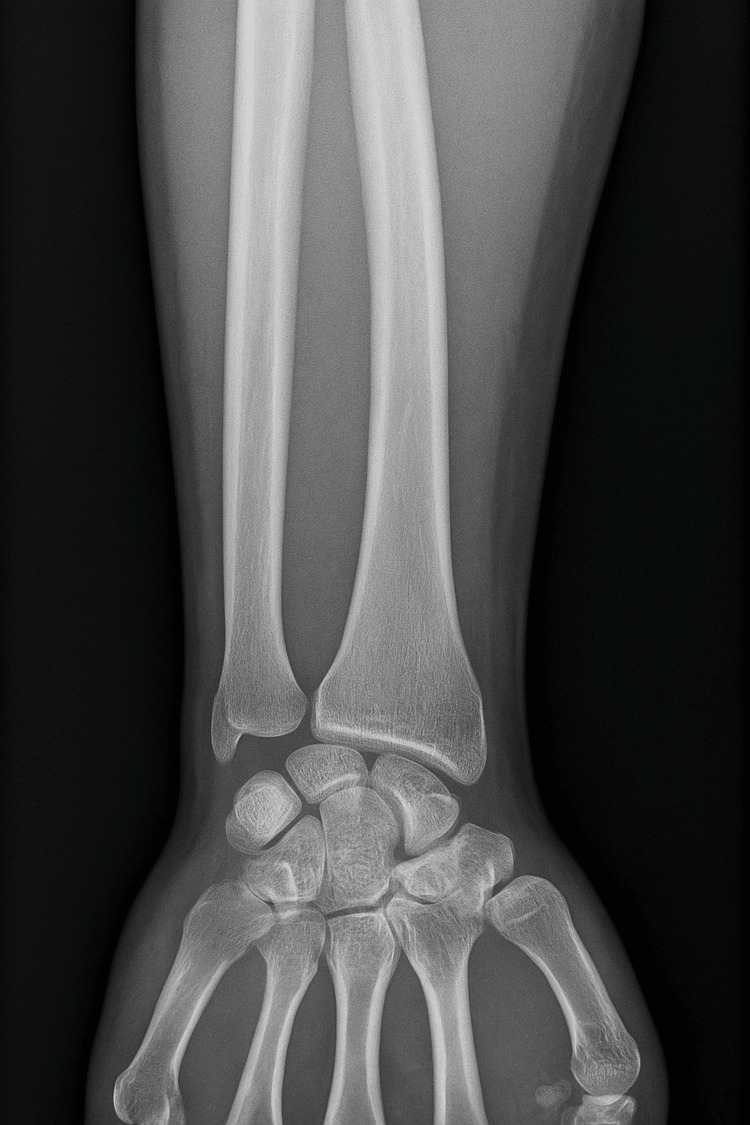
Conventional radiography of the right wrist and forearm. The posteroanterior view obtained following the trauma reveals no fractures or radio-opaque foreign bodies, confirming the absence of associated osseous injury.

A 27-year-old, right-handed, manual worker, with no significant past medical history, presented to our clinic two years after sustaining a 5 cm open wound to the anterolateral aspect of his right forearm’s distal third from an assault with a sharp weapon. Initial conventional radiography of the forearm (Figure 1) was unremarkable, showing no associated fractures or radio-opaque foreign bodies.

Initial clinical examination revealed a significant motor deficit. Muscle strength, evaluated using the Medical Research Council (MRC) scale, was 0/5, indicating no palpable contraction in the wrist flexor and superficial finger flexor muscle groups.

Surgical exploration was performed, which confirmed complete transection of the tendons corresponding to these muscles, including the palmaris longus, flexor carpi radialis, and the flexor digitorum superficialis tendons. These were surgically repaired with 4/0 non-absorbable filament sutures using Kessler knots. Postoperatively, the right upper limb was immobilized with a posterior splint in flexion, and the patient began early rehabilitative care.

Following several sessions of kinesiotherapy and occupational therapy, a follow-up muscle strength evaluation six months after the initial surgery showed significant recovery, with an MRC score of 4/5 in these muscle groups.

**Figure 2:**
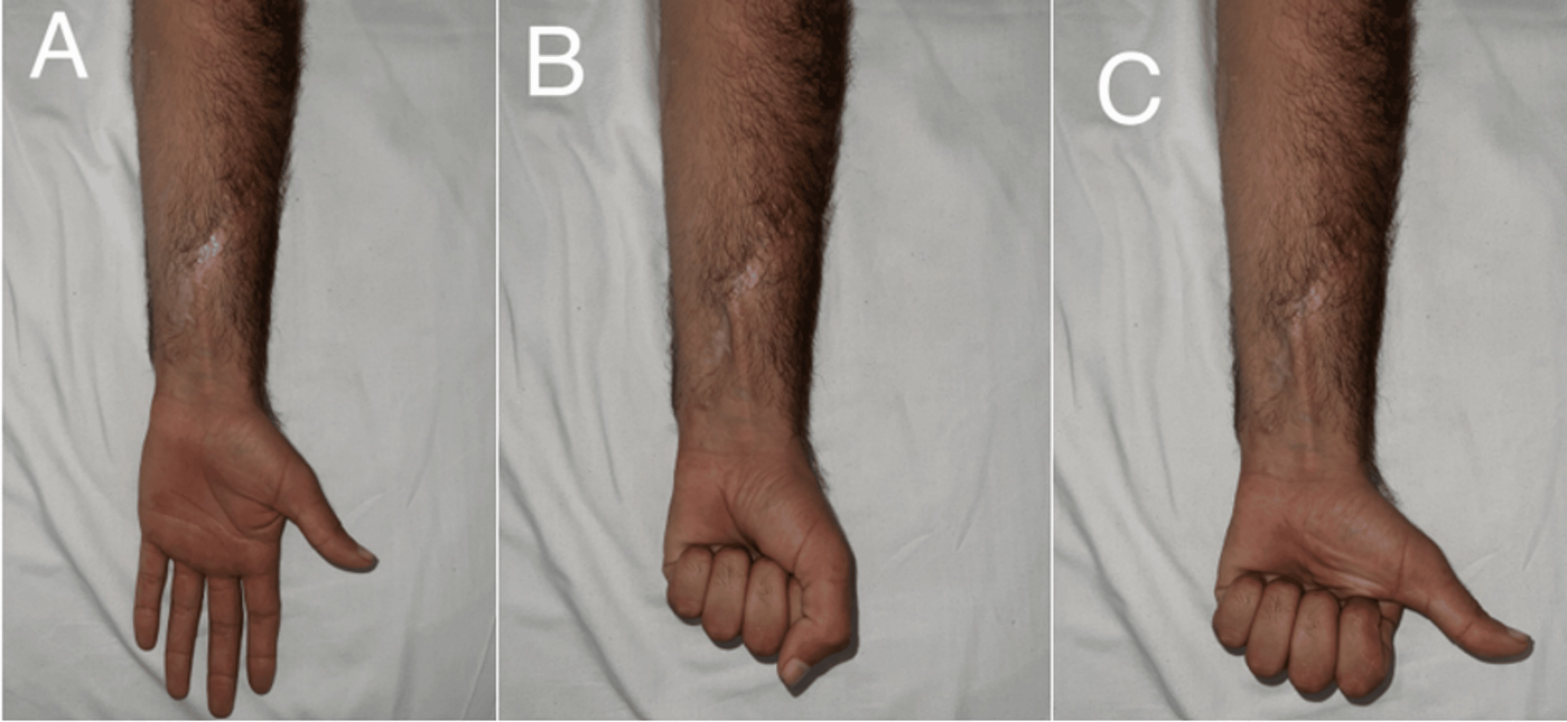
Clinical findings on the palmar surface of the right hand. A: Presence of a wound scar and associated skin atrophy, notably with preserved normal finger extension. B: Illustration of full, normal finger flexion, indicating the absence of motor weakness. C: The “hitchhiker’s sign” (thumb extension and abduction) is clearly positive, confirming no motor deficit of the radial nerve.

However, six months after the injury, the patient developed new symptoms. He reported severe neuropathic pain localized to the scar site (Figure 2A), radiating to the dorsal aspect of the thumb, index finger, and the dorsal thenar eminence. To objectively characterize this pain, the Douleur Neuropathique 4 Questions (DN4) scale was administered. It yielded a score of 7/10, a result based on the patient’s reports of burning sensations, pins and needles, and tingling, in addition to the presence of allodynia on clinical examination. The pain intensity was rated at 92/100 on a Visual Analog Scale (VAS). On examination, significant skin atrophy was noted at the scar site. Tinel’s sign was positive specifically over the SRN at this scar. Despite the severe sensory symptoms, motor testing was notably normal, with a clearly positive “hitchhiker’s sign” (thumb extension and abduction), thereby indicating intact motor function of the deep radial nerve and helping to exclude more proximal radial nerve pathologies (Figures 2B, 2C). No palpable swelling was noted.

The diagnostic workup included ENMG. For the SRN, the NCS were performed and yielded entirely normal results, with sensory nerve action potential amplitude, latency, and conduction velocity within the normal range when compared to the contralateral, unaffected side (Table 1). Consistent with the purely sensory nature of Wartenberg’s syndrome, the motor NCS and needle EMG components of the ENMG were also unremarkable, serving to exclude other potential motor neuropathies.

**Table 1:** Electroneuromyography results of the superficial radial nerve. The sensory nerve action potential (SNAP) values were within normal limits compared to the contralateral, unaffected side.

Parameter	Right (affected)	Left (unaffected)	Normal range
SNAP amplitude (µV)	35	38	>25
SNAP latency (ms)	2.1	2.0	<2.5
SNAP conduction velocity (m/s)	48	50	>45

**Figure 3:**
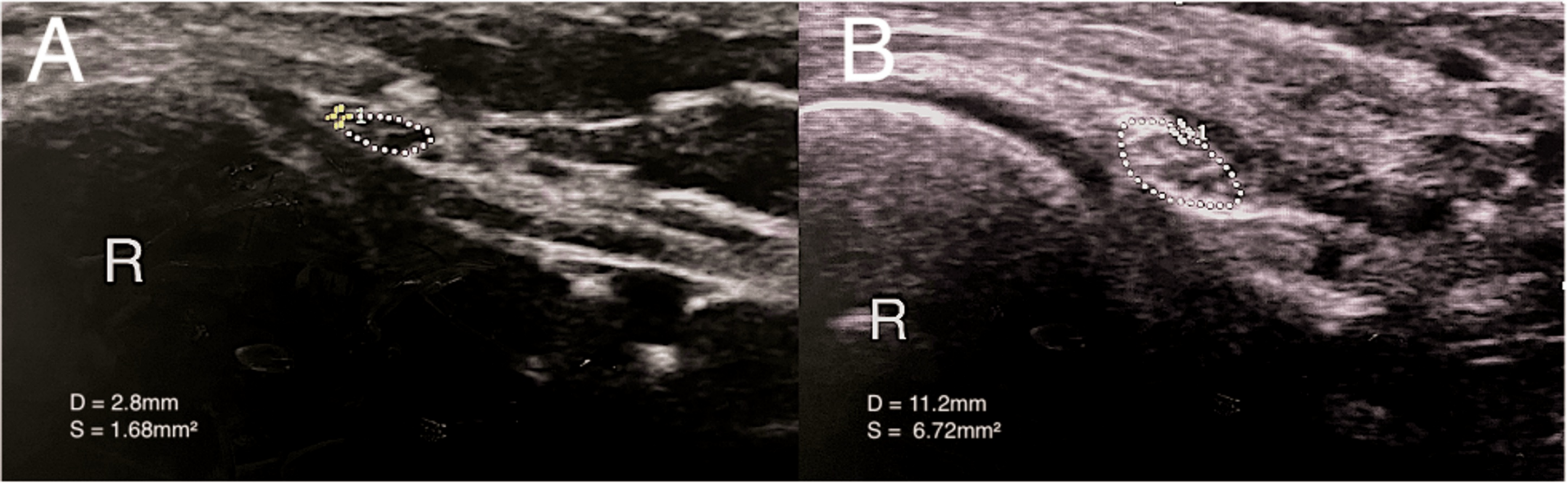
High-resolution ultrasonography images of the superficial radial nerve. A: Normal superficial radial nerve (SRN) on the contralateral side (R = radius), measuring 2.8 mm in diameter with a cross-sectional area (CSA) of 1.68 mm². B: Entrapped SRN on the affected side at the distal forearm scar site, showing marked enlargement (11.2 mm, CSA = 6.72 mm²) and hypoechoic appearance, consistent with entrapment neuropathy.

Subsequent high-resolution US of the forearm, however, objectively revealed a focal enlargement of the SRN at the scar site on the right side, measuring 6.72 mm² in cross-sectional area (CSA) compared to 1.68 mm² on the contralateral, unaffected left side (Figure 3). To further confirm the diagnosis and predict the response to interventional treatment, a diagnostic anesthetic block was performed: 5 mL of 0.75% bupivacaine was injected in a perineural distribution under US guidance. The test was positive, resulting in the complete disappearance of symptoms.

**Figure 4:**
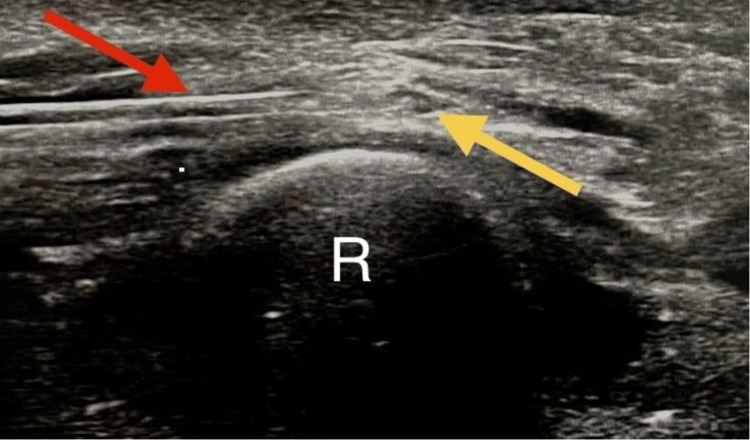
Ultrasonography-guided hydrodissection of the superficial radial nerve. An ultrasound image depicting the superficial radial nerve (yellow arrow) as it courses along the anterolateral border of the radius (R = radius). The image demonstrates the needle (red arrow) positioned for perineural hydrodissection of the nerve.

**Figure 5:**
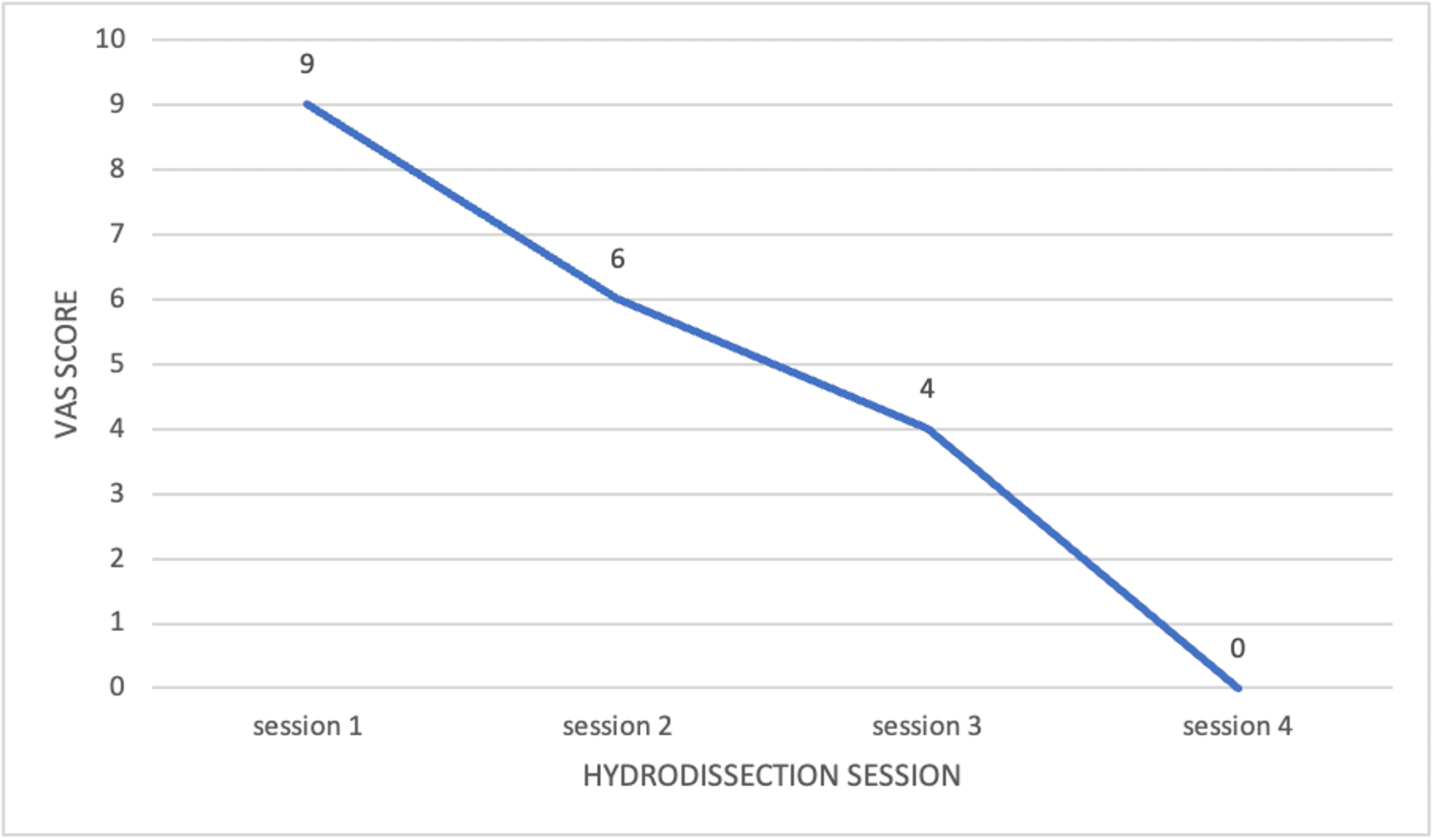
Visual Analog Scale (VAS) score reduction following superficial radial nerve hydrodissection.

Following this successful diagnostic block, US-guided hydrodissection of the SRN was performed at the entrapment site using a 5 mL perineural infiltration of 5% D5W solution (Figure 4). The patient received a total of four injections, spaced two weeks apart. This interventional treatment resulted in a sustained improvement, with the VAS score decreasing from an initial 9/10 to 0/10 after the fourth and final session. The progressive pain reduction is illustrated in Figure 5. This marked improvement led to the complete disappearance of symptoms and a successful return to his daily and professional activities.

Figure 5 illustrates the progressive decrease in pain intensity, as measured by the VAS, over the course of four US-guided hydrodissection sessions of the SRN. Initially, the patient reported severe pain with a VAS score of 9. Following the first hydrodissection session, a notable reduction to a score of 6 was observed. Subsequent sessions continued to show improvement, with a VAS score of 4 after the second session and complete pain resolution (VAS score of 0) after the fourth and final session. This downward trajectory highlights the effectiveness of hydrodissection in alleviating the pain symptoms of the SRN in this case.

## Discussion

The radial nerve, one of the largest branches of the brachial plexus, originates from the posterior cord with nerve fibers primarily from C6-C8, occasionally T1. It is susceptible to compression or injury at various points along its extensive anatomical course, leading to diverse etiologies. While the most frequent site of compression involves the posterior interosseous branch in the proximal forearm (affecting the supinator muscle area), issues can also arise more proximally (e.g., due to humeral fractures at the junction of the middle and proximal thirds) or more distally on the radial aspect of the wrist [7]. Wartenberg’s syndrome, or cheiralgia paresthetica, specifically involves damage to the superficial sensory branch of the radial nerve in the distal third of the forearm. While true entrapment neuropathy is rare at this level, traumatic injury is considerably more common given the nerve’s superficial course toward the wrist [8].

Wartenberg’s syndrome is characterized by pain and sensory disturbances primarily involving the dorsum of the hand and digits, which may, in some cases of more proximal entrapment, extend to the distal dorsolateral forearm [2]. Our patient, who developed severe, radiating neuropathic pain toward the dorsal thumb and index finger six months after direct forearm trauma, presents a clinical picture typical of this syndrome.

The diagnosis of SRN neuropathy often poses a challenge, particularly because conventional electrophysiological studies, such as ENMG, can yield normal results despite clear and debilitating clinical symptoms [3]. In our patient’s case, the ENMG was entirely normal, which is a frequent observation in pure sensory neuropathies or mild SRN compressions. This limitation of ENMG underscores the importance of a complementary diagnostic approach and highlights the crucial role of imaging.

In this context, high-resolution US proves to be a conclusive and indispensable diagnostic tool for the SRN [4,5]. It allows for direct and dynamic visualization of the nerve, offering precise anatomical details inaccessible by ENMG. US can objectively evaluate the morphology of the nerve, including its CSA, echogenicity, fascicular texture, and the presence of potential entrapment sites or adjacent masses [1,5]. In our patient, US definitively revealed focal enlargement of the SRN at the distal forearm scar site on the right side. This enlargement, measuring 6.72 mm² in CSA compared to 1.68 mm² on the contralateral, unaffected left side (Figure 3), directly indicated the nerve’s pathological response to compression by the adjacent scar tissue formed from the original wound. While the initial wound was not located directly on the dorsal hand, its position on the anterolateral aspect of the distal forearm was precisely along the SRN’s anatomical course, a well-recognized site for entrapment. This objective finding, together with the initial ENMG results that excluded motor involvement, and further supported by a positive diagnostic anesthetic block, allowed for the diagnosis of Wartenberg’s syndrome. The absence of sonographic signs of other etiologies (cysts, tumors, de Quervain’s tenosynovitis) also helped specifically guide the diagnosis. Although the mean CSA on transverse scan was only 2 mm² (range = 1-3 mm²) [4].

Regarding treatment, US-guided hydrodissection of the SRN with 5% D5W demonstrated remarkable efficacy in this case. Hydrodissection is a minimally invasive technique aimed at decompressing the nerve by injecting a fluid volume into the perineural space, thereby releasing the nerve from adhesions and reducing mechanical compression [6,9]. The choice of D5W as a hydrodissection agent is supported by growing evidence suggesting its benefits beyond simple mechanical decompression. Proposed mechanisms include the downregulation of the transient receptor potential vanilloid receptor-1 ion channel (implicated in chronic neuropathic pain) and a correction of perineural/intraneural glycopenia, as glucose is essential for neuronal metabolism [6,10]. The significant and progressive pain reduction observed in our patient (VAS score decreasing from 9/10 to 0/10 after four sessions) demonstrates the effectiveness of this interventional approach. This marked improvement led to a complete resolution of symptoms and a successful return to daily and professional activities, highlighting the therapeutic potential of D5W in managing entrapment neuropathies such as Wartenberg’s syndrome.

## Conclusions

This case report highlights a challenging presentation of Wartenberg’s syndrome (cheiralgia paresthetica) following trauma, characterized by severe neuropathic pain and notably normal ENMG findings. It unequivocally demonstrates the pivotal role of high-resolution US in providing a conclusive anatomical diagnosis by revealing nerve enlargement and entrapment. Furthermore, the successful and sustained resolution of symptoms following US-guided hydrodissection with D5W underscores its efficacy as a minimally invasive treatment option. This case reinforces the importance of integrating a thorough clinical evaluation with advanced imaging such as US, particularly when conventional electrophysiological studies are inconclusive, to achieve accurate diagnosis and effective management of SRN neuropathy.
